# An improved YOLOv11n-based method for high-precision detection of ginkgo fruits in complex natural environments

**DOI:** 10.3389/fpls.2026.1836867

**Published:** 2026-07-06

**Authors:** Zhenyang Lv, Zhentao Wang, Boyuan Tan, Yiqi Wang, Ruohan Wang, Sitong Liu, Guoqing Chen, Zhongbin Su, Hongbo Li

**Affiliations:** 1 College of Intelligent Science and Engineering, Northeast Agricultural University, Harbin, China; 2 Key Laboratory of Northeast Smart Agriculture Technology, Ministry of Agriculture and Rural Affairs, Harbin, China; 3 College of Mechanical and Electrical Engineering, Shihezi University, Shihezi, China; 4 College of Agricultural Equipment and Energy Engineering, Northeast Agricultural University, Harbin, China

**Keywords:** CED-YOLOv11n, ginkgo fruit detection, YOLOv11n improvement, object detection, smart agriculture

## Abstract

**Introduction:**

Ginkgo fruits in natural field environments are characterized by low color saliency, small target size, severe occlusion by branches and leaves, and complex illumination variations. These factors significantly reduce real-time detection accuracy and limit the applicability of existing object detection methods in practical agricultural scenarios.

**Methods:**

To address these challenges, this study proposes a real-time ginkgo fruit detection method based on an improved YOLOv11n framework. A multi-scenario dataset was constructed by collecting ginkgo fruit images under diverse lighting conditions, occlusion levels, and viewing angles, and data augmentation strategies were applied to improve sample diversity and model generalization. On this basis, a CFNet channel fusion module was embedded into the backbone network, a DynamicHead detection head was introduced to enhance multi-scale feature representation, and the original loss function was replaced with the Efficient IoU (EIoU) loss to improve bounding box regression accuracy. These improvements collectively form the proposed CED-YOLOv11n model, achieving a balanced optimization between detection accuracy and inference efficiency. The effectiveness of the model was validated through Grad-CAM visualization analysis, ablation studies, and comparative experiments with classical object detection models.

**Results:**

Experimental results show that the proposed CED-YOLOv11n achieves a precision of 94.6%, a recall of 85.4%, and a mean average precision (mAP) of 93.8% on the constructed ginkgo fruit dataset. In addition, the model is lightweight, with a parameter size of only 4.96 MB, and achieves an inference speed of 53.2 FPS, demonstrating strong real-time performance. Compared with mainstream object detection models such as DETR, Faster R-CNN, and YOLOv5, the proposed method achieves superior overall detection performance.

**Discussion:**

The results indicate that the proposed method effectively enhances the accuracy and efficiency of ginkgo fruit detection in complex natural environments. It provides technical support for the development of vision-based perception systems in intelligent ginkgo harvesting equipment and offers a reference for further optimization and integration of smart agricultural machinery systems.

## 1 Introduction

Ginkgo biloba, as an important tree species with both ecological and economic value, has been widely utilized in the pharmaceutical and food-processing industries due to its fruit properties (Nie et al., 2024; Mohammadi Zonouz et al., 2024). With the continuous expansion of the ginkgo industry, traditional manual harvesting methods have increasingly revealed limitations such as low operational efficiency, high fruit damage rates, and intensive labor requirements, making them incapable of meeting the demands of large-scale and standardized production. Consequently, harvesting equipment has gradually evolved toward intelligent and automated systems (Tang et al., 2020; Yang et al., 2021). In this context, vision-based fruit detection and localization have become critical prerequisites for achieving precise harvesting, and their performance directly affects operational efficiency and system stability (Bechar and Vigneault, 2016).

In practical orchard environments, ginkgo fruits are typically characterized by small target size, dense clustered distribution, and severe occlusion caused by branches and leaves. These factors collectively result in blurred object boundaries, weak feature saliency, and strong interference from complex backgrounds, which are widely recognized as the primary challenges restricting the improvement of visual detection performance in orchards (Tang et al., 2023; Huang et al., 2025). Early studies mainly relied on handcrafted features, such as color and texture, for target recognition. However, these methods exhibited poor robustness under varying illumination conditions and complex backgrounds (Hannan et al., 2007; Cubero et al., 2011). With the rapid development of deep learning, convolutional neural networks (CNNs) have substantially enhanced feature representation capabilities in agricultural vision tasks (Mohanty et al., 2016; Mureşan and Oltean, 2018). On this basis, one-stage object detection models, particularly the YOLO series, have been widely applied in fruit detection tasks because of their end-to-end architecture and high real-time performance (Hou et al., 2022; Wang et al., 2022). Nevertheless, existing studies have demonstrated that such methods still suffer from significant performance limitations in scenarios involving small objects, severe occlusion, and highly dense target distributions (Bochkovskiy et al., 2020).

To address the challenges of small targets, severe occlusion, and dense object distribution in complex scenarios, extensive studies have been conducted on multi-scale feature modeling, global relationship learning, and regression optimization. In the field of small-object detection, Lin et al. (2017) proposed the Feature Pyramid Network (FPN), which enhances the integration of high-level semantic information and low-level detailed features through a top-down multi-scale feature fusion strategy, thereby improving small-object detection performance to a certain extent. The Path Aggregation Network (PANet) further strengthened feature representation by optimizing feature transmission paths (Liu et al., 2018). Subsequently, the Bidirectional Feature Pyramid Network (BiFPN) introduced learnable weights to achieve efficient multi-scale feature fusion, providing a favorable balance between detection accuracy and computational efficiency (Tan et al., 2025). However, these methods mainly rely on static feature fusion mechanisms and still exhibit insufficient representation capability under complex backgrounds and weakly salient target conditions. To further enhance the modeling capability of object detectors in complex environments, Transformer-based detection methods were introduced into this field. Among them, the Detection Transformer (DETR) achieves global dependency modeling through a self-attention mechanism. However, its relatively low feature resolution and slow convergence speed limit its performance in small-object detection tasks (Carion et al., 2020). To address these limitations, Deformable DETR introduces a deformable attention mechanism to perform sparse sampling on multi-scale feature maps, thereby effectively improving the detection performance of small and densely distributed targets. Nevertheless, its relatively high computational complexity still restricts its applicability in resource-constrained agricultural scenarios (Zhu et al., 2020). In terms of occlusion handling, attention mechanisms have been widely adopted to enhance the perception capability of models toward critical regions. Among them, the Squeeze-and-Excitation (SE) module and the Convolutional Block Attention Module (CBAM) are the most representative approaches. The SE module enhances important feature responses through channel recalibration, but it focuses solely on channel-wise weight allocation and lacks the ability to model spatial positional information (Hu et al., 2018). CBAM further incorporates spatial attention based on channel attention, thereby improving the model’s ability to focus on target regions to some extent (Woo et al., 2018). However, both methods employ static attention mechanisms and cannot dynamically adjust weight distributions according to different scenarios, target scales, and task requirements. Consequently, their adaptability and representation capability remain limited when dealing with highly variable natural environments and targets with substantial scale variations (Dai et al., 2021). To improve dense-object detection performance, researchers have mainly focused on optimizing bounding-box regression losses. The Generalized Intersection over Union (GIoU) loss improves regression performance for non-overlapping targets by introducing the minimum enclosing box. However, it degenerates into the IoU loss when targets completely overlap and therefore fails to provide effective gradient information (Rezatofighi et al., 2019). Building upon this, the Complete Intersection over Union (CIoU) loss incorporates constraints on center-point distance and aspect ratio, thereby improving localization accuracy to a certain extent (Zheng et al., 2020). Nevertheless, it still fails to resolve the slow convergence problem caused by the coupling between aspect ratio and regression error (Du et al., 2021).

In summary, although existing studies have achieved certain progress in multi-scale feature fusion, attention mechanisms, and regression optimization, significant limitations still remain in complex agricultural scenarios. These limitations are mainly reflected in the insufficient representation capability for weakly salient small targets, the lack of dynamic modeling ability in detection heads, and the difficulty in balancing detection accuracy and computational efficiency under densely distributed target conditions (Mittal, 2024). Moreover, most existing methods have been developed for fruit types with relatively large sizes and clear spatial distributions. As a result, they are difficult to directly transfer to ginkgo fruit detection tasks characterized by small target size, dense clustered distribution, and severe occlusion. In addition, their relatively high computational cost further restricts real-time deployment on edge devices. Therefore, substantial research gaps still exist in ginkgo fruit recognition under complex natural environments, particularly in terms of small-object detection, occlusion handling, and lightweight deployment.

Based on the aforementioned issues, this study focuses on ginkgo fruit images collected under different harvesting scenarios and constructs a multi-scenario ginkgo fruit dataset. Building upon the YOLOv11n framework, the CFNet and Dynamic Head modules are introduced to improve the Backbone and Head architectures, respectively, while the Efficient Intersection over Union (EIoU) loss function is adopted. On this basis, an improved model, named CED-YOLOv11n, is proposed to achieve accurate ginkgo fruit detection in complex natural environments. The main contributions of this study are as follows: (1) the construction of a robust multi-scenario dataset for ginkgo fruit detection; (2) the design and implementation of a detection model that balances accuracy and real-time performance for ginkgo fruits; and (3) systematic validation of the proposed model through visualization analysis, ablation experiments, comparative experiments, and edge-device deployment experiments to comprehensively evaluate its detection performance.

## 2 Materials and methods

### 2.1 Data acquisition

#### 2.1.1 Image acquisition

**Figure 1:**
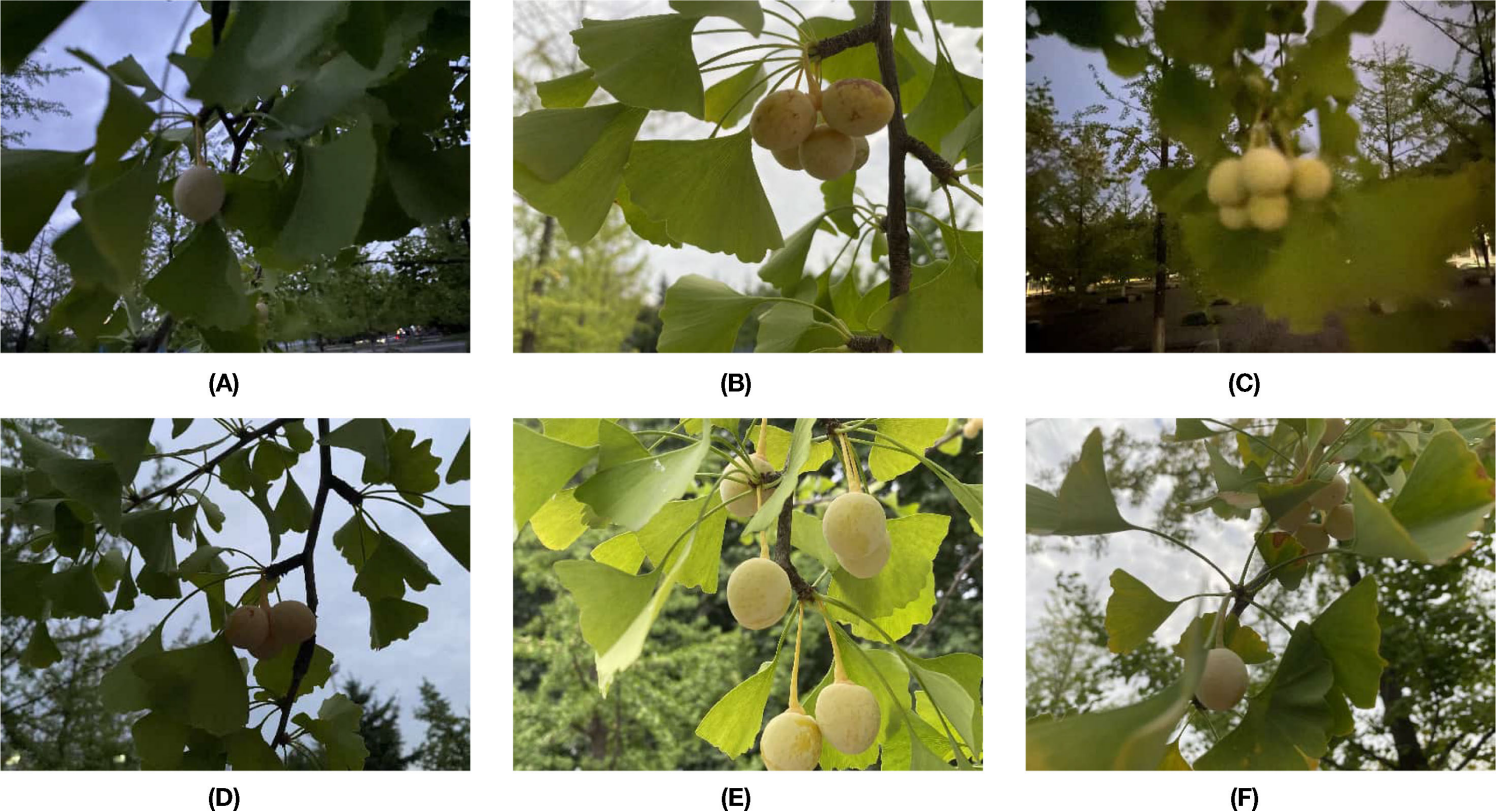
Ginkgo fruit images in different scenes. **(A)** Low light, **(B)** Fruit overlap, **(C)** Motion blur, **(D)** Backlight, **(E)** Normal light, **(F)** Foliage occlusion. Normal illumination images refer to images in which the overall brightness is relatively uniform, and there are clear color differences and edge contours between ginkgo fruits and leaf backgrounds, with relatively complete target texture information. Low-illumination images refer to images affected by cloudy weather, canopy occlusion, or acquisition time, in which the overall brightness is relatively low, the texture and edge information of fruits are weakened, and the contrast between fruits and leaf backgrounds is reduced. Backlit images refer to images in which the light source is located behind or at the rear side of the fruits; in such cases, the background region appears relatively bright, while the fruit regions exhibit shadows, partial underexposure, or abnormal contour contrast. For images that simultaneously contain multiple attributes such as low illumination, backlighting, occlusion, fruit overlap, or motion blur, they are categorized into the complex environment class.

In response to the practical requirements for automatic recognition of ginkgo fruits in natural growth environments, this study conducted continuous image acquisition during the ginkgo fruit maturation period (August to October 2024) in Harbin, Heilongjiang Province, China. During data collection, no artificial intervention was applied to the field environment, and all images were acquired under natural lighting conditions, so as to accurately reflect the appearance characteristics and spatial distribution patterns of ginkgo fruits in harvesting operations. The images were captured using a high-resolution color imaging device with a resolution of 3024 × 4032 pixels and stored in JPG format. No supplementary lighting or shading devices were used during the acquisition process, thereby preserving the influence of different weather conditions and time periods on fruit color, brightness, and texture characteristics. According to the size and morphological characteristics of ginkgo fruits, the shooting angle, position, and distance were appropriately adjusted to cover different scales and multi-view imaging conditions, while ensuring clear fruit details and retaining necessary background information. The collected scenes cover a variety of complex natural conditions, including sparse fruit distribution with relatively simple backgrounds, as well as dense fruit clusters with severe occlusion by branches and leaves (as shown in **Figure 1**). After manual screening, a total of 4,629 high-quality ginkgo fruit images were obtained and used as the original image dataset. The number of images collected under different environmental conditions is shown in **Table 1**. For images that contain multiple environmental attributes simultaneously, this study categorized them as a “complex environment” class to ensure accurate image classification. As shown in **Table 1**, the distribution of images under single environmental conditions is generally balanced, and images under complex conditions account for the largest proportion among all categories, providing a reliable data foundation for evaluating the robustness of the model in real-world unstructured environments.

**Table 1:** Number and corresponding proportion of ginkgo fruit images under different environmental conditions before and after data augmentation.

Environmental condition	Without data augmentation				With data augmentation				Proportion
	Training set	Validation Set	Testing set	Total	Training set	Validation set	Testing set	Total	
Low Light	397	132	132	661	1058	132	132	1322	14.28%
Fruit Overlap	356	119	119	594	950	119	119	1188	12.83%
Motion Blur	284	97	97	478	762	97	97	956	10.33%
Backlight	308	102	102	512	820	102	102	1024	11.06%
Normal Light	335	111	111	557	892	111	111	1114	12.03%
Foliage Occlusion	411	137	137	685	1096	137	137	1370	14.80%
Complex environments	686	228	228	1142	1826	228	228	2282	24.65%
Total	2777	926	926	4629	7406	926	926	9258	

“Complex environments” refers to images that simultaneously contain two or more scene elements. For example, if a single image contains both “Low Light” and “Foliage Occlusion” conditions, it is classified into the “Complex environments” category.

#### 2.1.2 Dataset annotation and augmentation

To construct a high-quality ginkgo fruit detection dataset, this study performed unified and standardized manual annotation on the collected image data. The annotation process was carried out using the open-source tool LabelImg, and all ginkgo fruit targets were labeled using rectangular bounding boxes and uniformly assigned to a single category, “Ginkgo”. For fruits without occlusion or with clear boundaries, bounding boxes were drawn as tightly as possible around the complete outer contour of the fruit. For targets occluded by leaves, branches, fruit stalks, or adjacent fruits, bounding boxes were defined based on the main visible region of the fruit, avoiding the inclusion of large invisible areas within the annotation box. In overlapping fruit scenarios, when adjacent fruits could still be distinguished based on contour, color, or partial boundary information, they were annotated as independent targets; when targets were severely blurred, had indistinguishable boundaries, or could not be reliably identified as ginkgo fruits, they were excluded from annotation. After annotation, an XML file was generated for each image, recording the category information and bounding box coordinates of fruit targets for subsequent supervised training of detection models. In natural orchard environments, ginkgo fruits are often affected by branch and leaf occlusion, fruit overlap, or partial visibility. Therefore, during annotation, priority was given to ensuring that bounding boxes covered the main visible region of the fruit to reduce annotation uncertainty under complex occlusion conditions. To minimize the impact of manual annotation errors on model training, all annotations were re-checked after initial labeling, and issues such as bounding box misalignment, scale anomalies, and duplicate annotations were corrected. The distribution of all labels in this dataset is shown in **Supplementary Figure 1**, from which it can be observed that the bounding boxes are mainly concentrated in the central region of the images and are generally small in size; most bounding box widths are distributed in the range of 0.05–0.2, while heights are mainly distributed in the range of 0.05–0.25. This indicates that the ginkgo fruit targets in the constructed dataset are predominantly small and medium-small objects. Meanwhile, the overall shape proportions of the bounding boxes are relatively stable, showing a clear positive correlation between height and width.

**Figure 2:**
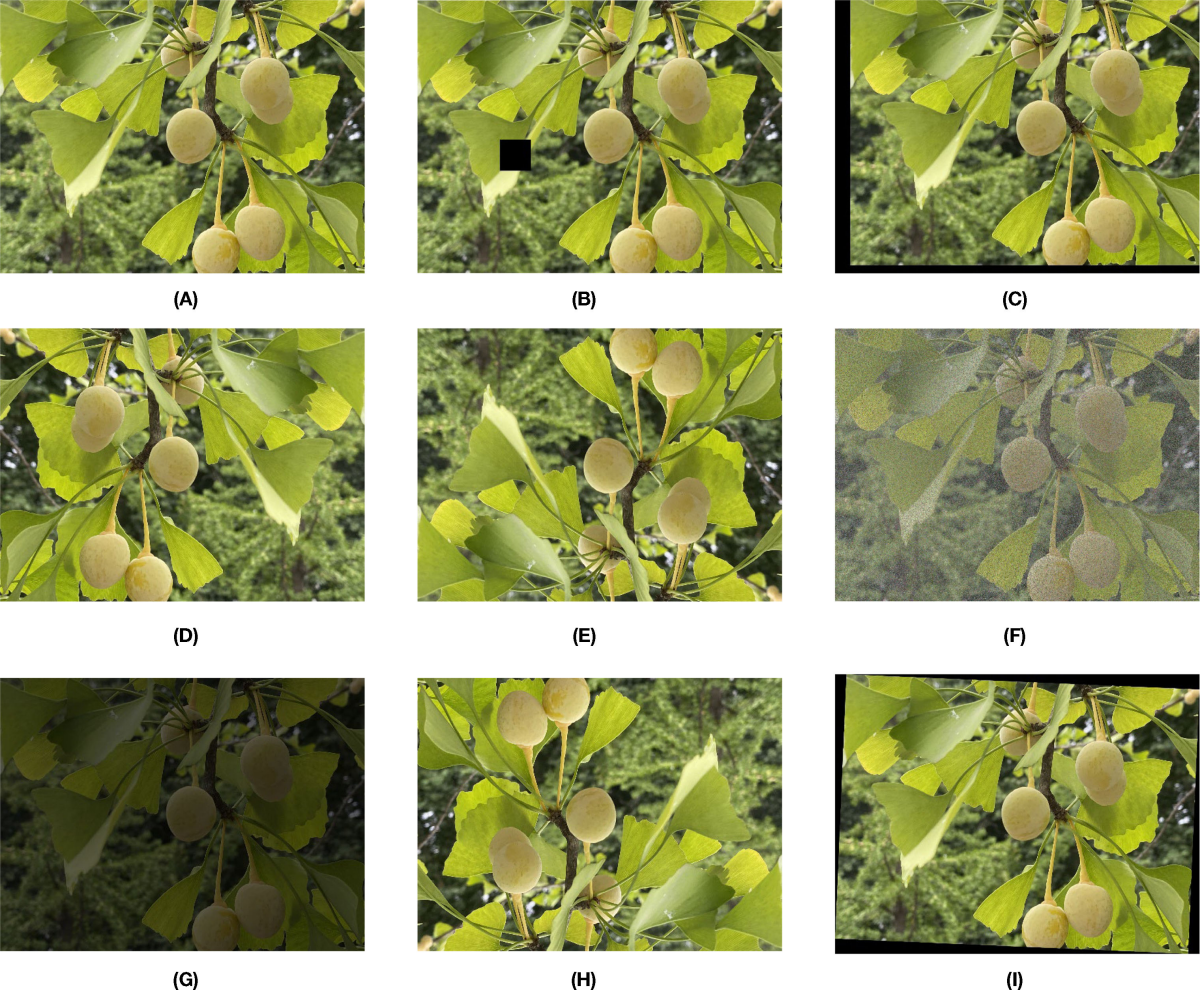
Ginkgo images after data augmentation. **(A)** Original image, **(B)** Image cutting, **(C)** Image translation, **(D)** Mirror image(left and right), **(E)** Mirror image(upper and lower), **(F)** Noise addition, **(G)** Brightness transformation, **(H)** Rotation angle(180°), **(I)** Rotation angle(arbitrary angle).

In addition, considering the variations in imaging pose and illumination conditions of the original samples, their feature distribution coverage is limited and insufficient to support the model’s generalization capability. Therefore, after annotation, this study introduced data augmentation strategies to expand the sample distribution, mainly including image rotation, flipping, cropping, and brightness adjustment, as shown in **Figure 2**. During the data augmentation process, bounding box coordinates were correspondingly adjusted to maintain consistency of annotation information. All data augmentation operations were applied only to the training set, while 926 original images were separately retained as the validation and test sets to objectively evaluate the model’s performance in real-world field conditions. Using the above augmentation strategy, the 2,777 training images were expanded to 7,406 images, resulting in a total of 9,258 ginkgo fruit images. Finally, the dataset was divided into training, validation, and test sets according to an 8:1:1 ratio, consisting of 7,406, 926, and 926 images, respectively, for model training and performance evaluation, as shown in **Table 1**.

### 2.2 Improvement of CED-YOLOv11n model

#### 2.2.1 Construction of the CED-YOLOv11n network

The YOLO series based on convolutional neural networks is a typical end-to-end object detection framework that formulates the detection task as a regression problem. Compared with two-stage object detection methods represented by R-CNN, YOLOv11 significantly improves inference efficiency while maintaining detection accuracy. The model is built upon a deep convolutional neural network, and it achieves sufficient multi-scale feature extraction and stronger feature representation capability by introducing efficient network architecture design and advanced data augmentation strategies, thereby effectively improving detection performance for objects of different scales as well as the overall robustness of the model. YOLOv11 provides five different model scales, including YOLOv11-n, YOLOv11-s, YOLOv11-m, YOLOv11-l, and YOLOv11-x, and each variant adopts a progressive configuration in terms of network depth and feature channel width to meet different computational resource constraints and application requirements. Among them, YOLOv11-n has the fastest inference speed and the smallest model size. Considering the requirements of real-time performance and lightweight deployment in practical scenarios, YOLOv11-n was selected as the baseline detection model in this study.

YOLOv11 object detection model adopts a typical three-stage architecture, consisting of Backbone, Neck, and Head as its fundamental components (Wang et al., 2021). In the input stage, the model applies an adaptive image scaling strategy to dynamically adjust the resolution and aspect ratio of input images, thereby preserving target geometric information as much as possible while reducing redundant computational cost. At the same time, an improved data augmentation mechanism is introduced, incorporating diverse image perturbations to enhance the model’s generalization ability in complex backgrounds, small-object scenarios, and occlusion conditions. In the Backbone design, YOLOv11 adopts CSPDarknet-11 as the main feature extraction network. This structure is based on the cross-stage partial (CSPNet) feature separation idea, and it reduces model parameters through a lightweight convolutional design while maintaining strong feature representation capability. Meanwhile, cross-stage residual connections are used to improve gradient propagation in deep networks. In the feature fusion stage, the Neck of YOLOv11 adopts a bidirectional progressive feature pyramid structure, which enables efficient interaction of multi-scale feature information through both bottom-up and top-down connections and alleviates the information loss problem commonly observed in traditional feature pyramid networks for small-object detection. The Head adopts a decoupled detection head design, which separately models classification and bounding-box regression tasks to reduce interference in multi-task learning, and it further incorporates a dynamic sample assignment strategy to improve sample matching quality during training and enhance overall detection performance.

**Figure 3:**
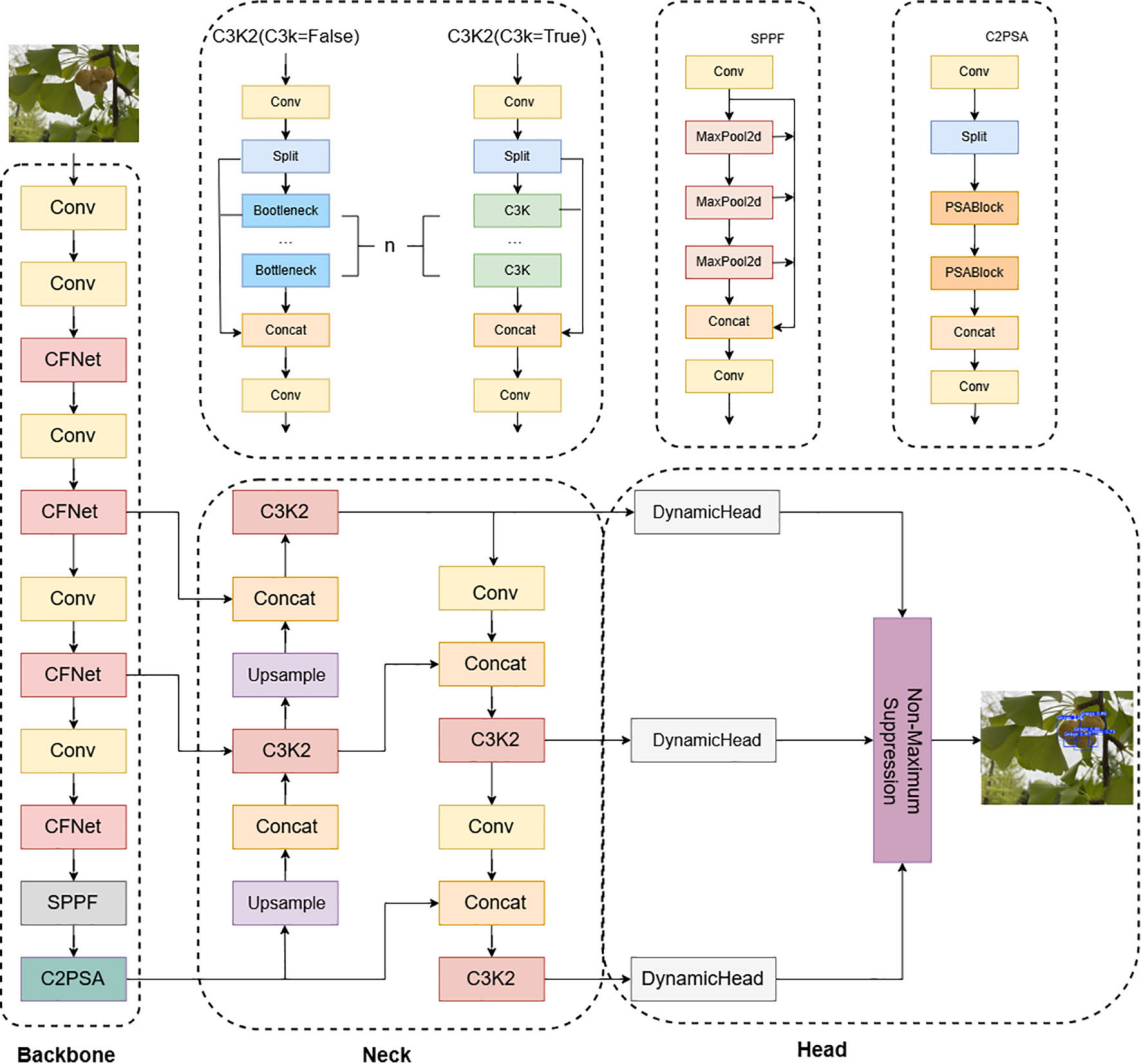
CED-YOLOv11n network structure diagram.

To address the three core challenges in ginkgo fruit detection under natural scenes, namely leaf occlusion, dense fruit distribution, and complex backgrounds, this study proposes targeted improvements to YOLOv11n. Specifically, a CFNet channel fusion module is embedded into the Backbone to enhance feature extraction capability for occluded targets. The localization loss in the original loss function is replaced with the Efficient Intersection over Union (EIoU) loss to improve bounding-box regression accuracy for densely distributed fruits. In addition, a Dynamic Head is introduced into the detection Head to enable adaptive detection of fruits at different scales and with varying levels of visibility. Based on these improvements, the proposed model, CED-YOLOv11n, is constructed, and its network architecture is illustrated in **Figure 3**.

#### 2.2.2 CFNet architecture

**Figure 4:**
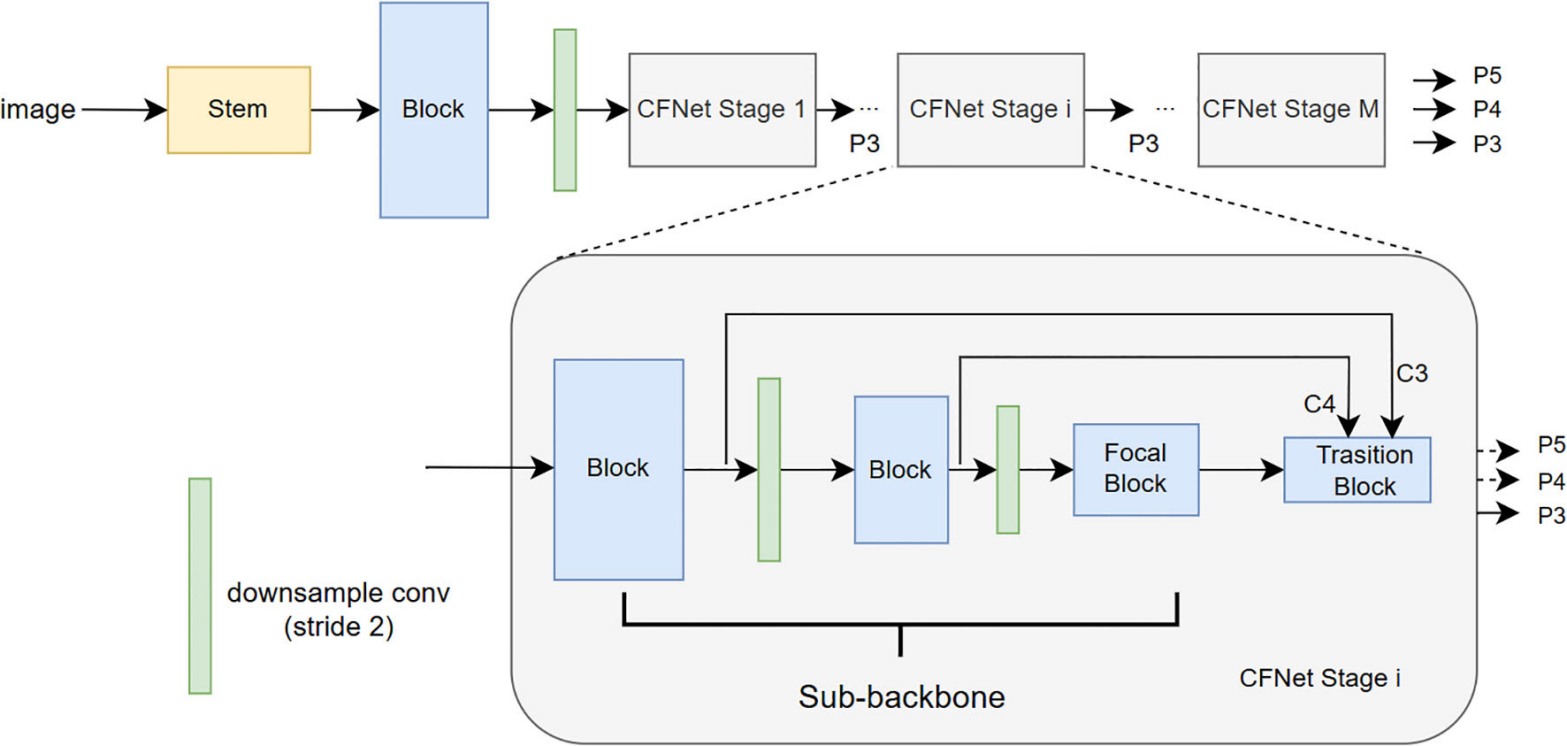
Network structure diagram of CFNet.

CFNet (Cascade Fusion Network) is a network architecture designed for dense prediction tasks, proposed by Zhang et al., 2023, as shown in **Figure 4**. Its core advantage lies in embedding feature aggregation operations into the backbone network, which enhances multi-scale feature fusion capability while maintaining a compact structure and computational efficiency, thereby addressing the limitations of insufficient feature fusion in traditional FPN architectures. The CFNet structure consists of a stem network (Stem), multiple convolutional blocks (Block), and M cascaded CFNet stages. Each CFNet stage includes a sub-backbone and a transition block, where the sub-backbone is constructed by alternately stacking standard blocks and focal blocks. In the entire network, only the stem network and downsampling convolutions reduce the spatial resolution of feature maps. This architecture enhances the depth of feature fusion through a cascade mechanism and expands the receptive field while controlling computational complexity, thereby improving the model’s representation capability for multi-scale objects.

CFNet consists of a multi-level hierarchical structure. In the backbone layer, a 3×3 convolution with a stride of 2 is applied, followed by LayerNorm and a GELU activation function. The initial continuous block layer is composed of several stacked basic blocks and is used to generate high-resolution features at 1/4 scale. In the CFNet stage layer, each stage contains a sub-backbone composed of multiple standard blocks and a focal block, as well as a transition block for feature aggregation. Each stage outputs multi-scale fused features with strides of 8, 16, and 32, respectively. Notably, only the shallowest features are propagated to the next stage. Finally, the multi-scale features generated by the last stage are jointly used for subsequent dense prediction tasks.

#### 2.2.3 Dynamichead detection head

**Figure 5:**
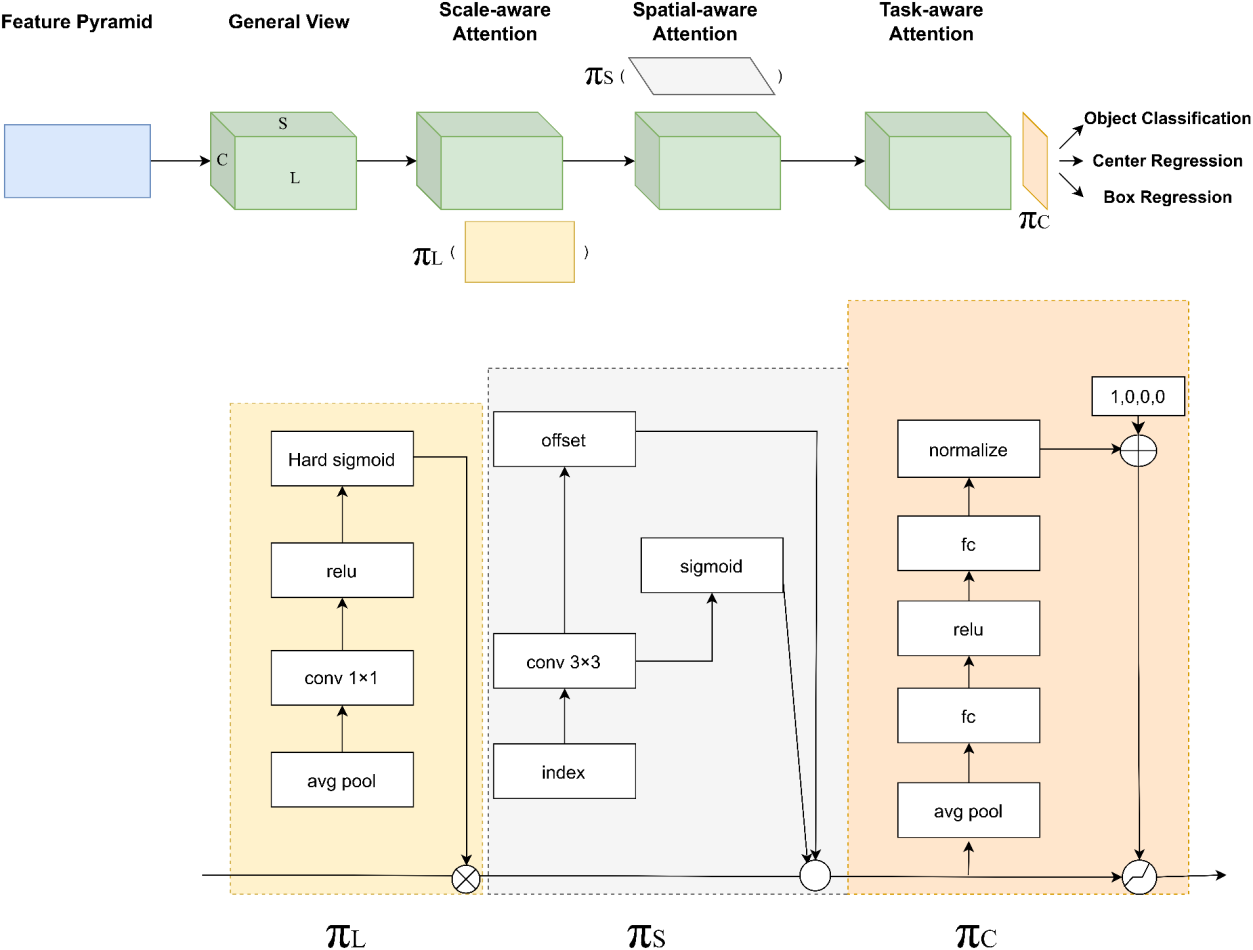
DynamicHead network structure diagram.

In natural environments, ginkgo fruits exhibit significant scale variation, and their appearance characteristics are unstable due to factors such as illumination changes, leaf occlusion, and background complexity. The detection head of the original YOLOv11n has a fixed structure, and its convolutional kernel parameters and receptive field cannot be adaptively adjusted according to feature quality, making it difficult to adequately handle variations in fruit size and appearance inconsistency. To address these issues, this study introduces a Dynamic Head into the Head component of YOLOv11n, which enhances the model’s ability to perceive complex fruit features through a multi-dimensional attention-driven adaptive mechanism. Dynamic Head decomposes conventional self-attention operations into sequential scale-aware, spatial-aware, and task-aware attention, thereby improving the representational capacity of the detection head without significantly increasing computational cost (Dai et al., 2021). The network architecture is illustrated in **Figure 5**.

DynamicHead consists of three main components: a feature input adaptation layer, a multi-dimensional attention fusion module, and a feature output enhancement layer. The feature input adaptation layer first receives the multi-scale feature pyramid output from the Neck and performs channel alignment and tensor transformation, mapping features at different scales into structurally consistent three-dimensional tensors, thereby providing a unified input for subsequent sequential attention. The multi-dimensional attention fusion module is the core of DynamicHead, which sequentially applies scale-aware attention (π_l), spatial-aware attention (π_s), and task-aware attention (π_c) to perform fine-grained feature refinement. The feature output enhancement layer is responsible for deeply integrating the features after dynamic attention fusion and producing enhanced feature representations for bounding-box regression and classification prediction. This layer preserves the cross-scale modeling advantages brought by dynamic attention, enabling the detection head to achieve stronger generalization ability and stability in complex scenarios.

#### 2.2.4 EIoU loss function

In object detection model training, the loss function is used to quantify the discrepancy between predicted bounding boxes and ground-truth boxes and plays a critical role in both convergence speed and localization accuracy. In the conventional YOLOv11 model, the CIoU loss is employed, however, CIoU tends to suffer from gradient saturation and unstable convergence when regressing small or densely distributed targets with large aspect ratio differences. To address this issue, the Extended IoU (EIoU) loss function was adopted in this study to replace CIoU. EIoU explicitly models and decouples the positional, width, and height deviations in bounding box regression, allowing each regression component to be independently optimized and thereby reducing mutual interference among different error terms. This approach enables the model to obtain more stable gradient information during training and significantly improves responsiveness to variations in bounding box dimensions, ultimately enhancing localization accuracy and overall detection performance in complex scenarios (Du et al., 2021). The calculation formula for EIoU is as follows (Equation 1):

(1) $$I_{EIoU}=1-IoU+\frac{({b_{x}-b_{x}^{g})}^{2}}{w_{c}^{2}}+\frac{({b_{y}-b_{y}^{g})}^{2}}{h_{c}^{2}}+\frac{({w-w_{g})}^{2}}{w_{c}^{2}}+\frac{({h-h_{g})}^{2}}{h_{c}^{2}}$$

In the formula, *IoU* represents the intersection over union between the predicted box and the ground-truth box, *b_x_* and *b_y_*​ denote the horizontal and vertical coordinates of the predicted box center, while *w* and *h* correspond to the width and height of the predicted box, *w_c_*and *h_c_* represent the width and height of the smallest enclosing box that contains both the predicted and ground-truth boxes.

### 2.3 Software and hardware configuration

All detection models in this study were run under the same hardware and software configurations, and a unified data preprocessing pipeline and parameter settings were adopted to minimize interference from environmental and other external factors, thereby ensuring the accuracy and reliability of the experimental results. The experiments were conducted on a computing platform equipped with an Intel i7-14700HX processor with a base frequency of 2.4 GHz, 16 GB DDR5 RAM, and an NVIDIA GeForce RTX 4070 discrete GPU. The deep learning experimental environment was built based on Python 3.9 and PyTorch 1.13.1, with CUDA 11.8 and cuDNN 8.6 used for GPU-accelerated computation. To achieve a good balance between detection accuracy and inference speed, and to meet the requirements of real-time recognition in natural environments, an input image resolution of 640 × 640 was adopted for both training and testing. During model training, the initial learning rate was set to 0.01 to ensure fast convergence in the early training stage while avoiding oscillations in parameter updates. The stochastic gradient descent (SGD) optimizer was used to ensure stable training performance. The weight decay coefficient was set to 0.937 to constrain parameter updates, reduce the risk of overfitting, and improve the model’s generalization ability in complex orchard environments. The total number of training epochs was set to 200 to ensure that the model could sufficiently learn the feature distribution of ginkgo fruits under varying illumination, occlusion, and blur conditions. Meanwhile, the batch size was set to 16 to improve the randomness of gradient estimation while maintaining training efficiency.

### 2.4 Ablation and comparative experiment

In this study, the CFNet and DynamicHead modules are respectively integrated into the Backbone and Head to extract image features, while the Efficient Intersection over Union (EIoU) loss function is adopted to improve the classical YOLOv11n model. To fully demonstrate the advantages of the proposed improvements and evaluate their mutual interactions, ablation experiments were conducted to compare and analyze the performance of the detection network before and after modification. Ablation studies are commonly used in complex neural networks and refer to a method of understanding network behavior by selectively removing components and evaluating performance changes. This approach has been widely applied in deep learning research (Wang et al., 2023). The models used in the ablation study and their corresponding modules are presented in **Table 2**.

**Table 2:** Improved models of the ablation test.

Improved models	Improved modules			
	YOLOv11n architecture	CFNet	EIoU	DynamicHead
YOLOv11n	√	—	—	—
Model1	√	√	—	—
Model 2	√	—	√	—
Model 3	√	—	—	√
Model 4	√	√	√	—
Model 5	√	√	—	√
Model 6	√	—	√	√
CED-YOLOv11n	√	√	√	√

To further validate the performance superiority and scene adaptability of the proposed CED-YOLOv11n in Ginkgo fruit detection tasks, this study selected eight representative mainstream algorithms in the field of agricultural object detection as comparative baselines, including two-stage detection methods, one-stage detection methods, and Transformer-based detection architectures. Specifically, the compared models included EfficientDet (Liu et al., 2025), DETR (Li et al., 2025a), Faster R-CNN (Wang et al., 2025a), RetinaNet (Xiao et al., 2026), YOLOv5 (Zhuo et al., 2025), YOLOv10 (Tan et al., 2024), YOLOv11 (Yang et al., 2025), and YOLOv12 (Tian, et al., 2026). Among them, YOLOv5, YOLOv10, YOLOv11, and YOLOv12 adopted their lightweight n-version models, namely YOLOv5n, YOLOv10n, YOLOv11n, and YOLOv12n (Chen et al., 2026), respectively. To ensure the fairness and reliability of the comparative experiments, all models were subjected to the same data preprocessing pipeline and dataset splitting strategy. In addition, all models were trained and tested under identical hardware and software environments as well as the same hyperparameter settings, and quantitative evaluation was conducted using identical performance metrics. Meanwhile, no additional inference acceleration or model compression techniques were applied to any of the compared models, thereby ensuring the objectivity and comparability of the experimental results.

### 2.5 Evaluation metrics

To comprehensively evaluate the performance of the proposed improved model CED-YOLOv11n in ginkgo fruit detection tasks, this study adopted two commonly used categories of evaluation metrics in the field of object detection, namely detection performance metrics and model complexity metrics. Detection performance included Precision, Recall, F1-Score, and mean Average Precision (mAP). Model complexity included key parameters such as weight size and frames per second (FPS). These metrics enabled a comprehensive assessment of the model in terms of accuracy, stability, computational efficiency, and deployment feasibility. Specifically, Precision represents the proportion of correctly predicted ginkgo fruit samples among all samples predicted as fruits, reflecting the reliability of the model predictions. Recall indicates the proportion of actual ginkgo fruits successfully detected by the model and is used to measure the missed detection rate. F1-Score is the harmonic mean of Precision and Recall, which comprehensively reflects the balance between precision and recall. Mean Average Precision (mAP) is used to evaluate the overall detection performance across all classes and confidence thresholds (Ju et al., 2024). In this study, mAP@0.5 (with the IoU threshold set to 0.5) was adopted as the primary evaluation metric to ensure the comparability and stability of the results.

In addition, to evaluate the practicality of the model in real deployment scenarios, this study introduces model weight size (MB) and real-time detection speed (FPS). The weight size reflects the storage resources required by the model, while FPS indicates the inference speed of the model. Both metrics are closely related to the requirements of real-time detection in agricultural orchard applications (Mittal, 2024). The calculation methods for all the aforementioned evaluation metrics are defined as follows (Equations 2–5):

(2) $$Precision=\frac{TP}{TP+FP}$$

(3) $$Recall=\frac{TP}{TP+FN}$$

(4) $$F1=\frac{2\times Precision\times Recall}{Precision+Recall}$$

(5) $$mAP=\frac{1}{c}\sum_{i=1}^{c}AP_{i}$$

In the formulas, *c* represents the total number of classes, *TP* denotes the number of samples whose true class and predicted class are both positive, *FP* denotes the number of samples predicted as positive but actually negative, and *FN* denotes the number of samples whose true class is positive but were not detected by the model, *APi* represents the average precision of the i-th class.

## 3 Results analysis

### 3.1 Grad-cam visualization analysis of different improved models

**Figure 6:**
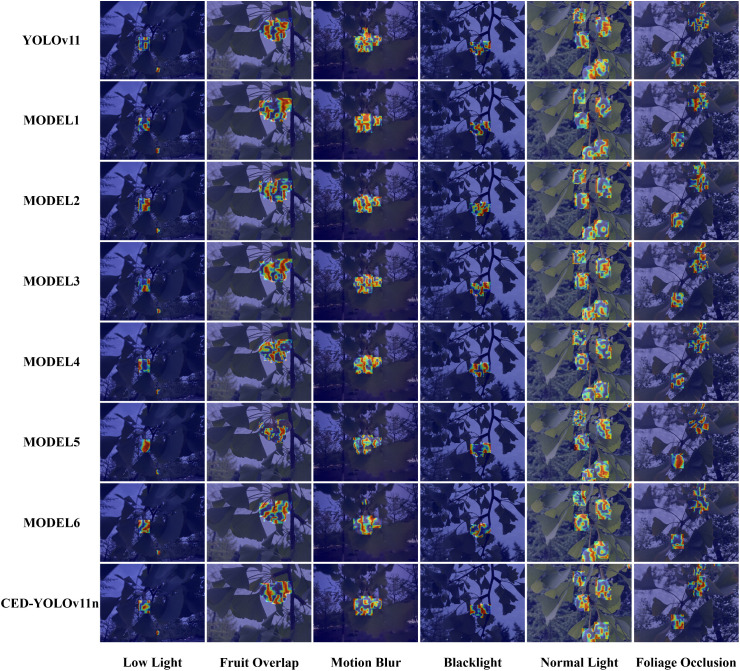
Comparison of Grad-CAM visualization results of different improved models in different scenarios.

To qualitatively evaluate the perceptual capability of the improved model in capturing key features of ginkgo fruits as well as its decision-making robustness, this study employs Gradient-weighted Class Activation Mapping (Grad-CAM) to visualize network feature responses (Selvaraju et al., 2017). By analyzing the saliency maps of different improved models under complex conditions such as illumination variation, fruit overlap, occlusion, and blur, the spatial attention distribution patterns during the model’s prediction process can be intuitively revealed, and the visualization comparison results are shown in **Figure 6**.

The experimental results indicate that the original YOLOv11n model exhibits a significant attention dispersion effect when extracting ginkgo fruit features. In most scenarios, the heatmaps show broadly distributed high-response regions. Some background regions are even misidentified as fruit targets, resulting in non-target activation points. This phenomenon reflects the insufficient spatial semantic focusing capability of the baseline model and its tendency toward feature misalignment. With the progressive introduction of key improvement modules, the heatmaps of different models exhibit differentiated trends of attention optimization. Model 1 effectively reduces background interference. However, its thermal response for small fruits under low-light conditions remains weak. In occlusion scenarios, the completeness of heat coverage for partially occluded fruits is still insufficient. Model 2 produces more compact heatmap boundaries in motion-blur scenarios and effectively eliminates background interference. Nevertheless, some occluded fruits still fail to generate sufficient activation responses. Compared with the fused attention regions of the baseline model, Model 3 incorporating DynamicHead can initially distinguish heat boundaries between overlapping fruits, significantly improving discriminability. However, attention dispersion still occurs under low-light conditions. Although the dual-module configurations (Model 4, Model 5, and Model 6) achieve performance improvements in specific scenarios, they still exhibit single-dimensional limitations in scene adaptability. For example, Model 5 achieves more complete heat coverage in occlusion scenarios than Model 1, while Model 6 provides clearer boundary discrimination in overlapping scenes than Model 3. However, Model 4 still shows insufficient response intensity under low-light conditions. Finally, the fully integrated CED-YOLOv11n model combining CFNet, EIoU, and DynamicHead achieves the best spatial alignment performance across all test scenarios. Its heatmap activations remain consistently concentrated at the geometric center of ginkgo fruits, while the response weights in non-target regions are significantly reduced. These results confirm that the proposed architecture fundamentally enhances feature discrimination capability in complex backgrounds.

Based on the growth characteristics of ginkgo fruits and real-world operational environments, this study used Grad-CAM heatmaps to analyze the differences in feature responses of different models under multi-source interference. Under static challenges such as fruit occlusion, overlap, and complex illumination, the original YOLOv11n and the comparison models exhibited varying degrees of limitations. Model 3 was capable of distinguishing heat boundaries of overlapping fruits and correcting attention misalignment under backlit conditions. However, it still lacked effective responses in heavily occluded regions and for small targets under low-light conditions. Model 1 improved response intensity in low-light environments. Nevertheless, it remained insufficient in boundary clarity under backlighting and in coverage completeness in occluded regions. In contrast, CED-YOLOv11n demonstrated superior scene adaptability. It not only enabled continuous high-response activation for internal and overlapping fruits under leaf occlusion, but also maintained stable and comprehensive heat coverage under both low-light and backlit conditions. This effectively overcame the drawbacks of weak responses and background shift observed in traditional models. When further focusing on dynamic scenarios, Model 1 showed better background suppression than other single-module configurations. However, CED-YOLOv11n performed more prominently. Its heatmaps remained consistently and tightly aligned with the fruit body, without diffusion toward the background or morphological distortion caused by blur artifacts. Overall, benefiting from the synergistic effects of CFNet cross-scale feature fusion, DynamicHead multi-dimensional modulation, and the EIoU mechanism, the proposed model achieved a transition from globally diffused feature attention to precise local target focus. This provides solid support for high-precision real-time detection in complex operational environments.

### 3.2 Ablation experiment results of different improvement modules

**Figure 7:**
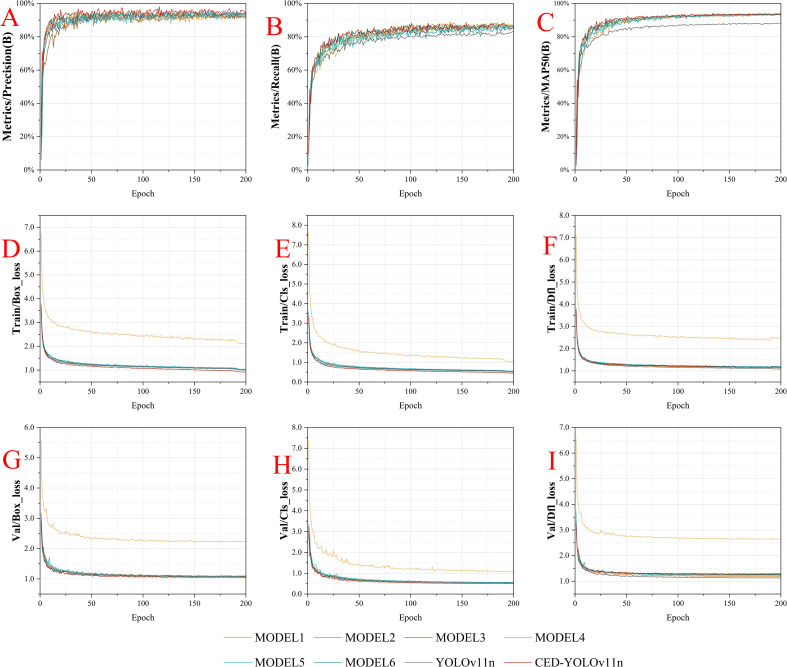
The performance curves of different improved network models. **(A)** Precision, **(B)** Recall, **(C)** mAP, **(D)** Boundary box loss of training set, **(E)** Classification loss of training set, **(F)** Confidence loss of training set, **(G)** Boundary box loss of validation set, **(H)** Classification loss of validation set, **(I)** Confidence loss of validation set.

To further investigate the specific contributions of the proposed CFNet channel fusion, EIoU-based bounding box regression optimization, and DynamicHead multi-dimensional attention mechanism to model performance, a systematic ablation study was designed. Using the original YOLOv11n as the baseline architecture, differentiated comparison model groups were constructed by incrementally embedding individual modules and combining multiple modules in a coupled manner. This section quantitatively evaluates the effectiveness of each improvement module and their synergistic effects in the ginkgo fruit detection task from multiple perspectives, including detection accuracy (Precision, Recall, and mAP), model complexity (Parameters and FLOPs), and real-time performance (FPS) (Chai et al., 2025), and the detailed quantitative metrics and training dynamics curves are presented in **Table 3**, **Figure 7**, respectively.

**Table 3:** The comparison results of detection performance of different improved network models.

Models	Precision (%)	Recall (%)	mAP (%)	FLOPs	Parameters	Weight size (MB)	FPS
YOLOv11n	91.4	82.6	88.3	6.40 × 10^9^	2.59 × 10^6^	5.22	**56.3**
Model 1	92.0	86.9	93.5	6.80 × 10^9^	**2.45 × 10** ^6^	**4.94**	54.1
Model 2	92.4	86.9	**94.0**	6.40 × 10^9^	2.59 × 10^6^	5.22	48.3
Model 3	92.3	**87.2**	93.3	6.40 × 10^9^	2.59 × 10^6^	5.22	50.3
Model 4	93.1	84.8	93.5	6.80 × 10^9^	**2.45 × 10^6^**	4.96	46.2
Model 5	93.2	84.5	93.6	**6.80 × 10^9^**	**2.45 × 10^6^**	4.96	52
Model 6	93.7	86.0	93.4	6.30 × 10^9^	2.59 × 10^6^	5.22	48.4
CED-YOLOv11n	**94.6**	85.4	93.8	6.80 × 10^9^	**2.45 × 10** ^6^	4.96	53.2

Bold values indicates the optimal values in each column.

Analysis of the performance curves in **Figure 7** shows that all single-module improvements can significantly enhance the accuracy of YOLOv11n, thereby verifying the effectiveness of the proposed strategies. As shown in **Table 3**, after embedding CFNet into the backbone network (Model 1), mAP increases from 88.3% to 93.5%, representing an improvement of 5.2 percentage points. The recall reaches 86.9%. Meanwhile, both the parameter count and model weight slightly decrease. This achieves a balance between lightweight design and accuracy improvement and strengthens the feature representation of dense ginkgo fruits. Compared with the traditional FPN-based improvement strategy proposed by Meng et al. (2021), CFNet better alleviates small-object feature loss while maintaining model compactness. Therefore, it achieves dual optimization of accuracy and lightweight design. After replacing the loss function with EIoU (Model 2), mAP reaches 94.0%, which is 5.7 percentage points higher than the baseline model. This represents the best performance among all single-module configurations. The EIoU loss function independently models the width and height errors of bounding boxes. It effectively avoids the gradient saturation problem of CIoU in dense clustered fruit scenarios. As a result, precision increases to 92.4%, significantly improving localization robustness. Model 3 achieves a recall of 87.2%, with mAP and precision reaching 93.3% and 92.3%, respectively. This effectively reduces missed detections. Its built-in sequential attention mechanism adaptively perceives multi-scale variations in ginkgo fruit morphology. It dynamically adjusts the receptive field and enhances the detection capability and recognition stability of fruits under complex illumination and varying scales. This finding is consistent with the results reported by Guan et al. (2025). Their GC-Faster R-CNN model introduced a hybrid attention mechanism that effectively alleviated feature ambiguity caused by multi-scale objects and high-similarity interference, thereby significantly improving recall. Overall, these results confirm that attention-driven feature enhancement strategies are crucial for addressing missed detections of multi-scale small objects in unstructured agricultural environments. The experimental results of Models 1–3 further demonstrate differentiated optimization effects. Specifically, EIoU (Model 2) contributes most significantly to overall accuracy improvement (mAP). CFNet (Model 1) achieves the best trade-off between enhanced feature extraction and model compression. DynamicHead (Model 3) performs best in reducing missed detections and improving recall. This complementary behavior provides a solid basis for integrating the three modules to construct combined models (Models 4–6) and the final CED-YOLOv11n model.

With the progressive integration of the proposed improvement modules, the model achieves a better balance between detection accuracy and overall robustness (Zhang et al., 2025a). The combined improved models (Models 4–6) consistently maintain precision above 93.1%. The final improved model, CED-YOLOv11n, reaches a precision of 94.6%, representing an improvement of 3.2 percentage points over the baseline model. Although its mAP (93.8%) is slightly lower than that of Model 2, it demonstrates a more favorable trade-off between lightweight design and inference speed. The model weight is only 4.96 MB, while the inference speed reaches 53.2 FPS. Therefore, it can fully satisfy the real-time detection requirements of practical agricultural applications. In terms of computational efficiency, CED-YOLOv11n maintains FLOPs at 6.80 × 10^9^. While ensuring high-precision detection performance, it successfully achieves lightweight deployment of the model. This provides favorable conditions for hardware adaptation in edge computing terminals.

**Figure 7** clearly illustrates the dynamic differences and performance stratification among the models during the training convergence process. As shown in **Figures 7A-C**, CED-YOLOv11n consistently maintains a significant leading advantage throughout training. Its Precision, Recall, and mAP curves continuously increase and gradually stabilize within the optimal performance range. In contrast, the accuracy curves of Model 1, Model 2, and Model 3 remain noticeably lower than those of CED-YOLOv11n. These models each incorporate only a single improvement module. Specifically, Model 1 with CFNet enhances feature extraction capability for occluded targets. However, its performance improvement remains limited because it lacks dynamic adaptation mechanisms and loss function optimization. Meanwhile, the baseline YOLOv11n exhibits relatively low overall performance. This further highlights the significant performance gap between single-module improvements and multi-module synergistic optimization. The loss curves in **Figures 7D-I** further reveal the internal training dynamics of the models. Model 1 maintains relatively high loss values throughout the entire training process. After an initial decline, its convergence speed slows down significantly. This phenomenon is mainly attributed to the increased model complexity. The multi-scale feature cascade fusion introduced by CFNet substantially enlarges the parameter learning space. As a result, the model requires more iterations to complete feature adaptation. Although the convergence speed decreases, the loss curves remain smooth and stable throughout training. No abnormal fluctuations or divergence are observed. This indicates that the increased training cost limits rapid performance improvement to some extent. Nevertheless, CFNet still effectively enhances feature representation. This effectiveness is reflected in the final accuracy, which remains slightly higher than that of the baseline model. Unlike the limitations of single-module configurations, CED-YOLOv11n achieves simultaneous improvements in both training efficiency and generalization capability. The localization loss (Val/Box_loss) and classification loss (Val/Cls_loss) on the validation set are both reduced to the lowest levels. In addition, the gap between validation loss and training loss is minimized. These results fully demonstrate the synergistic effectiveness of the EIoU loss function and the DynamicHead mechanism. The introduction of these two components effectively offsets the additional computational burden caused by CFNet. The three modules form complementary advantages. This design not only mitigates the risk of overfitting but also comprehensively improves model accuracy, stability, and generalization capability while maintaining stable training behavior.

### 3.3 Comparison of performance with classical detection models

The results of the comparative experiments in terms of detection accuracy, model complexity, real-time performance, and scene adaptability are presented in **Table 4**. Overall, different detection architectures exhibit significant performance variations in the ginkgo fruit detection task. CED-YOLOv11n achieves the highest precision among all models, reaching 94.6%. This value is improved by 2.6%, 1.8%, 3.2%, and 1.6% compared with YOLOv5n, YOLOv10n, YOLOv11n, and YOLOv12n, respectively. These results indicate that the proposed model can effectively recognize ginkgo fruits under various scenarios. This performance advantage is likely attributed to the synergistic effects of CFNet, DynamicHead, and the EIoU loss function. These components enable effective feature extraction under complex conditions such as illumination variation and branch occlusion. They also reduce false detections and localization deviations caused by boundary confusion in dense fruit regions. In contrast, the Transformer-based DETR model achieves a precision of 58.3% and an mAP of 56.3%. This represents the poorest performance among all compared models. This is mainly because DETR focuses on modeling long-range dependencies and end-to-end detection. However, it fails to effectively attend to target regions in dense small-object scenarios. As a result, missed detections and inaccurate localization frequently occur (Nsumba et al., 2026). Faster R-CNN exhibits relatively strong region proposal capability, with a recall of 81.3%. However, its precision is only 68.5%. This may be attributed to the generation of redundant proposals under complex natural backgrounds in the two-stage detection pipeline. These redundant proposals lead to accumulated localization errors (Li and Lee, 2025b). RetinaNet and EfficientDet achieve relatively high precision values but lower mAP and real-time performance. This indicates that these models still have limitations in multi-scale feature utilization and complex background discrimination (Wu et al., 2025). Among the YOLO-based models, YOLOv5n, YOLOv10n, and YOLOv12n all demonstrate relatively strong performance. YOLOv10n achieves the highest mAP of 94.6%, which is slightly higher than that of CED-YOLOv11n. This result indicates its strong overall detection capability. However, CED-YOLOv11n achieves a better balance among precision, model size, and inference speed. Its model weight is only 4.96 MB, and its inference speed reaches 53.2 FPS. These characteristics make it more suitable for deployment on edge devices in resource-constrained orchard environments.

**Table 4:** Comparison of detection results between CED-YOLOv11n and classical deep learning models.

Models	Precision (%)	Recall (%)	mAP (%)	F1-score	Weight size (MB)	FPS
EfficientDet	92.8	78.7	84.5	0.85	25.64	13.5
DETR	58.3	66.1	56.3	0.56	158.89	6.44
Faster R**-**CNN	68.5	81.3	82.9	0.75	108.17	4.42
RetinaNet	90.2	73.2	79.9	0.81	138.92	11.99
YOLOv5	92.0	87.7	93.5	**0.92**	13.71	19.69
YOLOv10	92.8	**88.4**	**94.6**	0.91	15.75	30.67
YOLOv11n	91.4	82.6	88.3	0.87	5.22	**56.3**
YOLOv12	93	85.9	93.8	0.89	5.22	35.46
CED-YOLOv11n	**94.6**	85.4	93.8	0.90	**4.96**	53.2

Bold values indicates the optimal values in each column.

In terms of model computational capability and deployment adaptability, parameters scale, computational load, and inference speed determine the practical application potential of a model on orchard mobile harvesting platforms, embedded terminals, and edge devices (Zhan et al., 2026). As shown in **Table 4**, the CED-YOLOv11n model has a weight of only 4.96 MB, which is the lowest among all compared models. It corresponds to only 4.6% and 3.1% of the weights of Faster R-CNN and DETR, respectively. These results demonstrate a strong lightweight advantage. This advantage is mainly attributed to the compact structure of the YOLOv11n backbone. It is also attributed to CFNet, which enhances feature fusion capability without significantly increasing computational cost. Therefore, the deployment cost on edge devices is effectively reduced. It is worth noting that traditional two-stage models and Transformer-based models generally exhibit larger model sizes and lower inference speeds. This may be due to the reliance of Faster R-CNN on region proposal generation and secondary classification–regression processes. These processes lead to higher computational complexity (Tong et al., 2025). In addition, DETR requires global attention modeling. This makes efficient deployment difficult on resource-constrained agricultural devices (Yu et al., 2025). In terms of real-time performance, the introduction of DynamicHead enhances dynamic feature representation. However, it leads to a certain reduction in inference speed compared with lightweight baselines. Despite this, CED-YOLOv11n still achieves 53.2 FPS. This is sufficient to meet the requirements of real-time ginkgo fruit perception in field operations. Therefore, the results indicate that CED-YOLOv11n achieves a better balance among high accuracy, lightweight design, and real-time performance. This makes it more suitable for precise ginkgo fruit detection in natural orchard environments.

**Figure 8:**
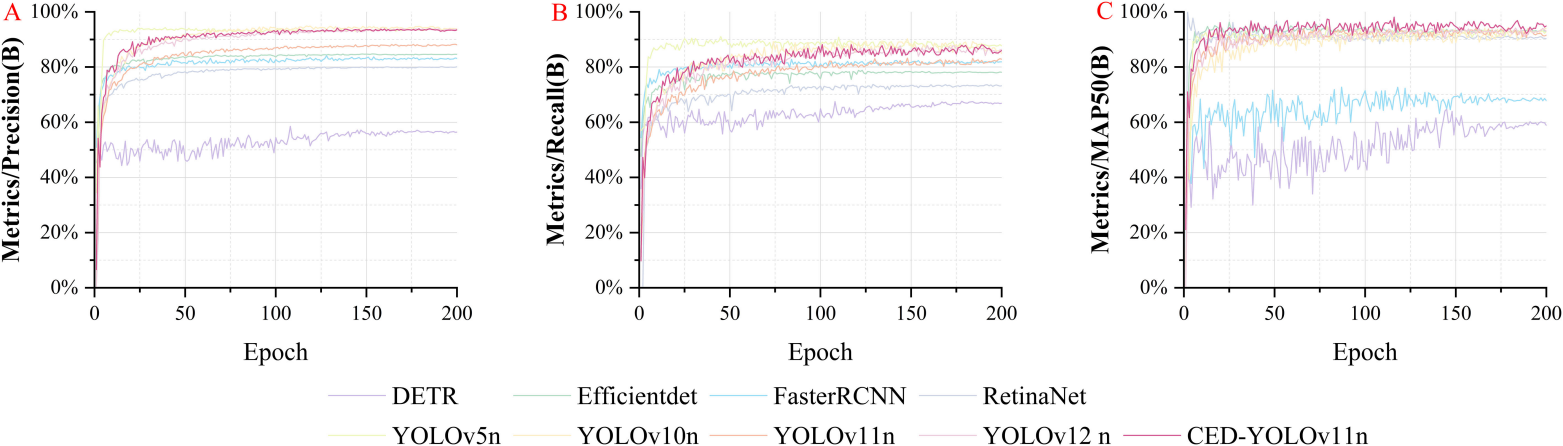
The comparison results with the performance curve of the classical target detection model. **(A)** Precision, **(B)** Recall, **(C)** mAP.

The corresponding training dynamics comparison curves are shown in **Figure 8**. These curves include three sub-curves: Precision, Recall, and mAP. They provide an intuitive basis for dimension-wise analysis of the training dynamics of different algorithms. From the Precision metric, CED-YOLOv11n exhibits the best performance. It shows a rapid increase in the early training stage. Driven by the synergistic effect of the improved modules, it achieves efficient feature learning. It also enters the stable phase earlier than other models. Throughout training, it maintains the highest precision level. In contrast, DETR remains in a low-precision range. It also shows frequent fluctuations and poor stability. Faster R-CNN shows a relatively slow increase in precision. It ultimately achieves a significantly lower value than CED-YOLOv11n. YOLO-based models achieve final precision values close to CED-YOLOv11n. However, their convergence efficiency is still insufficient. From the perspective of Recall, different models exhibit distinct behaviors. YOLOv10n and YOLOv5n achieve slightly higher final recall than CED-YOLOv11n. All three models remain stable in the later training stage. No obvious fluctuations are observed, which indicates good stability. In contrast, DETR maintains consistently low recall. Although its fluctuation decreases in later stages, it still shows a significant gap compared with other models. It also fails to meet detection requirements. Faster R-CNN converges faster than CED-YOLOv11n. However, it does not reach a high-level recall range. It also enters a performance plateau in the mid-to-late training stage. No further improvement is observed, which reflects limitations in its recall optimization capability. As the core metric reflecting overall detection performance, the mAP curves further validate the advantages of CED-YOLOv11n. It rapidly breaks through performance bottlenecks in the early stage. It reaches a high-level steady state with a significantly faster convergence rate. Throughout training, it shows no performance degradation or severe oscillation. This demonstrates excellent feature adaptability and stability. In contrast, DETR remains in a low mAP range with persistent fluctuations. Even after partial stabilization, it still performs poorly. Faster R-CNN enters a plateau phase in the middle stage, with minimal further improvement. EfficientDet and RetinaNet fluctuate within a mid-to-high range. However, both still show a noticeable gap compared with CED-YOLOv11n, indicating their limited overall detection performance.

### 3.4 Analysis of detection performance variability across different scenarios compared with classical models

**Figure 9:**
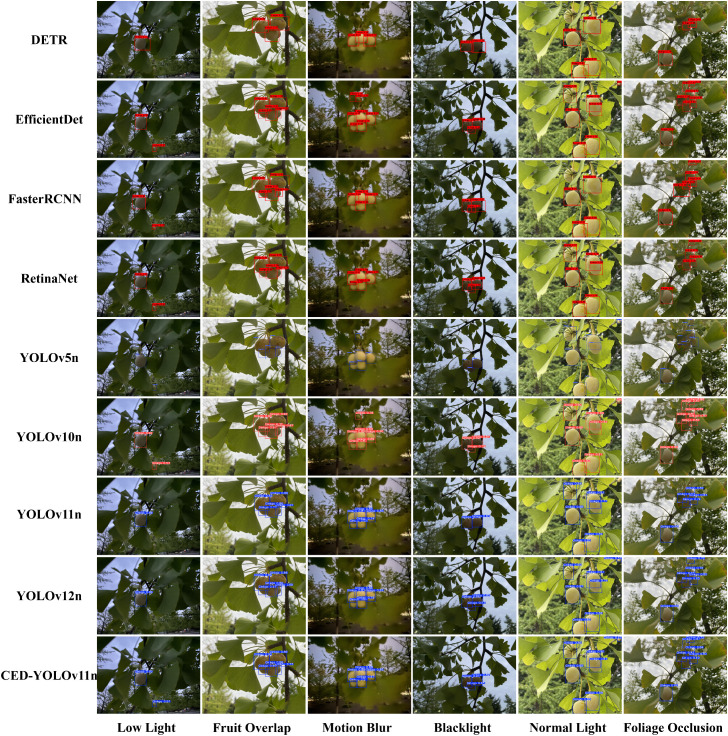
Comparison of recognition effects between CED-YOLOv11n and classic models in different scenarios.

To further validate the detection robustness of the proposed CED-YOLOv11n model in complex orchard environments, this study conducted visual comparative experiments under six typical challenging scenarios, including low light, fruit overlap, motion blur, backlighting, normal illumination, and leaf occlusion. The comparison models include DETR, EfficientDet, Faster R-CNN, RetinaNet, and YOLO series models (v5n, v10n, v11n, and v12n), and the qualitative results are shown in **Figure 9**.

The results in **Figure 9** show that different models exhibit significant performance variations under complex scenarios. In low-light conditions, where the color and brightness contrast between Ginkgo fruits and leaf backgrounds is markedly reduced, several models suffer from decreased confidence, localization shifts, or missed detections. Among them, the DETR model is entirely influenced by its global attention mechanism and struggles to stably focus on fruit regions when object size is small and background textures are complex, resulting in fewer detection boxes and noticeable target omissions. This may be attributed to the fact that its global attention mechanism is easily distracted by branches, sky regions, and background textures, making it difficult to effectively extract weakly salient features of Ginkgo fruits. Faster R-CNN and RetinaNet are able to detect part of the fruits, however, they are constrained by insufficient responsiveness to low-saliency targets in multi-stage feature fusion and redundant region proposals in the two-stage pipeline, respectively, leading to varying degrees of bounding box misalignment, missed detections, and low confidence scores. For EfficientDet, although some detected fruit bounding boxes exhibit relatively high confidence, most detections remain low-confidence, accompanied by numerous false positives and missed detections, indicating that this model is not well-suited for Ginkgo fruit recognition tasks. Notably, YOLOv5n demonstrates the most stable performance among the classical YOLO comparison models, as it can reliably localize Ginkgo fruits across all six scenarios, with relatively high confidence scores and fewer false and missed detections. This indicates that the CSP backbone and FPN–PAN feature fusion architecture in YOLOv5n still provide strong baseline feature representation capability for small-object detection of Ginkgo fruits (Zhang et al., 2025b). In particular, under normal illumination, fruit overlap, and leaf occlusion conditions, YOLOv5n is able to preserve fruit contour information effectively, which may be attributed to its mature multi-scale detection head and relatively stable anchor matching mechanism. However, YOLOv5n still shows limitations under low-light, backlit, and blurred conditions. When the contrast in color and brightness between fruits and leaf backgrounds decreases, the feature pyramid tends to suffer from insufficient semantic information or localization deviation during small-object feature propagation, thereby affecting its stable recognition performance for Ginkgo fruits.

YOLOv10n demonstrates outstanding performance in motion blur scenarios, enabling more complete fruit detection. This is consistent with the observations in **Table 3**, where YOLOv10n achieves the highest recall among all models, indicating that it has relatively stronger robustness under severe dynamic blur interference. Among all detection models, CED-YOLOv11n achieves the best overall performance, as it not only maintains high-confidence detection under normal illumination and fruit overlap conditions, but also remains stable in target detection under challenging conditions such as low light, backlighting, and leaf occlusion, while producing more compact bounding boxes and fewer missed detections. This improvement is mainly attributed to the synergistic effects of CFNet, DynamicHead, and EIoU, where CFNet enhances inter-channel information interaction and multi-scale feature fusion, thereby alleviating fine-detail loss of small objects during deep downsampling (Li et al., 2025c); DynamicHead introduces adaptive attention modulation across scale, spatial, and task dimensions, improving the model’s responsiveness to illumination variation, occlusion, and object scale fluctuations (Li et al., 2025d); and EIoU further strengthens joint constraints on bounding box center distance, width–height consistency, and overlap area, enabling more precise localization under clustered fruits and blurred boundaries (Ju et al., 2023). Therefore, although YOLOv5n performs best among classical YOLO-based methods, CED-YOLOv11n still outperforms YOLOv5n and other comparison models in detection stability and adaptability to complex scenarios, demonstrating superior comprehensive performance for Ginkgo fruit detection in natural orchard environments.

### 3.5 Edge-device deployment feasibility and performance evaluation

**Figure 10:**
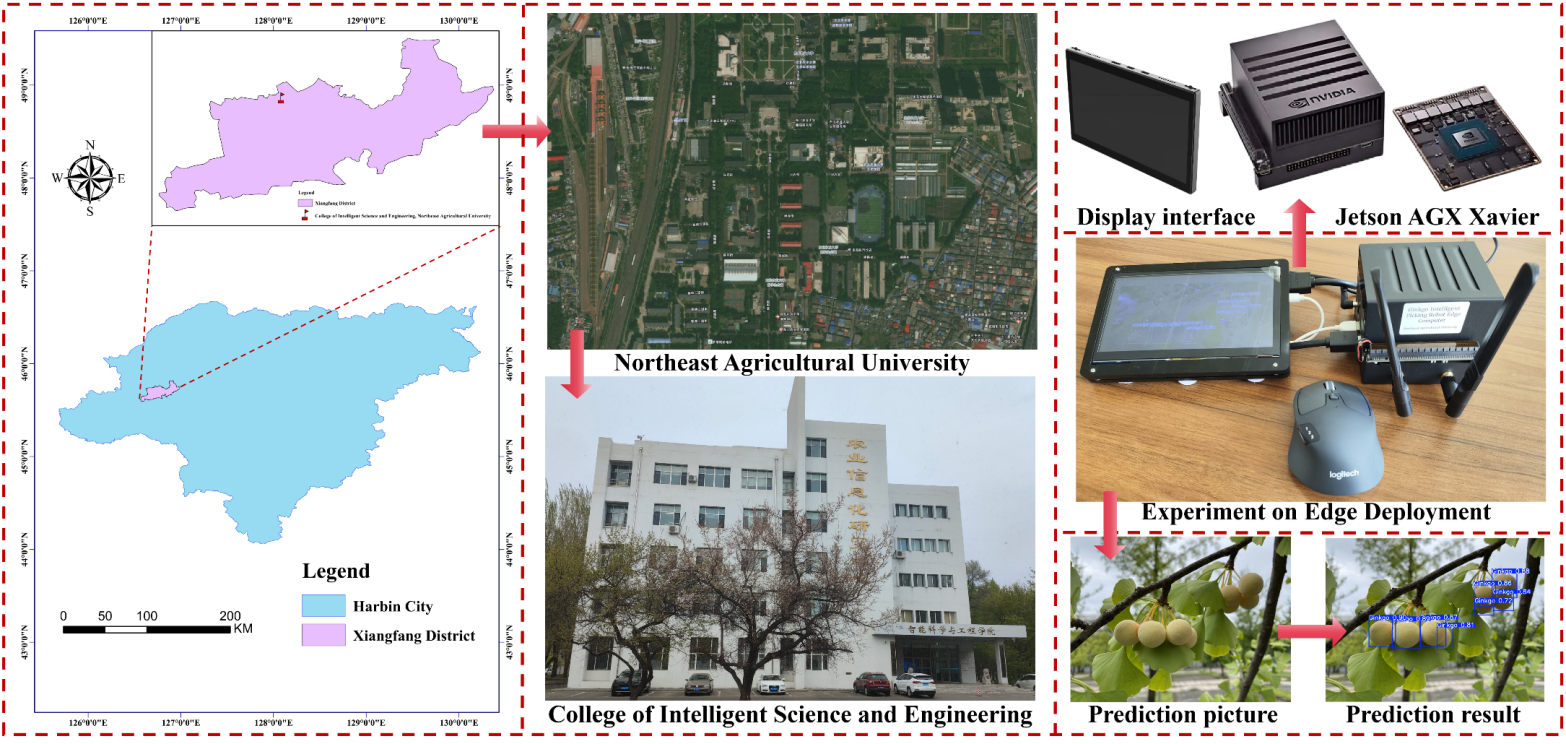
Location and results of the improved CED-YOLOv11n model deployment on the NVIDIA Jetson AGX Xavier platform.

To comprehensively evaluate the practical applicability of the proposed CED-YOLOv11n model under resource-constrained conditions, this study employs the NVIDIA Jetson AGX Xavier as a representative edge-computing platform (CPU: 8-core ARM v8.2 64-bit CPU with 8 MB L2 and 4 MB L3 cache; GPU: NVIDIA Volta architecture equipped with 512 CUDA cores and 64 Tensor Cores; AI computing capability: 32 TOPS INT8), thereby constructing an embedded inference testing environment (Mwitta et al., 2024), and the related experiments were conducted at the College of Intelligent Science and Engineering, Northeast Agricultural University. The system architecture and experimental results are shown in **Figure 10**, **Table 5**, respectively. Under a unified experimental configuration, the optimal weights obtained during training were deployed to both YOLOv11n and CED-YOLOv11n models, and on-device inference tests were conducted using a self-built Ginkgo fruit dataset. To improve the reliability of the experimental results, each model was subjected to ten repeated performance tests, and the detailed results are provided in **Supplementary Table 1**. By comparing the detection performance and runtime efficiency of the two models on a low-computing-power platform, their adaptability in real-world application scenarios was evaluated. The experimental results demonstrate that CED-YOLOv11n exhibits good stability and adaptability in embedded environments, and is capable of accurately completing Ginkgo fruit detection tasks, with all detection confidence scores consistently above 86%, indicating strong feature modeling capability. Meanwhile, its average inference speed reaches 23.74 FPS, demonstrating satisfactory real-time processing capability under limited hardware resources. Overall, CED-YOLOv11n achieves a favorable balance between accuracy and speed, validating its deployment feasibility and engineering application potential on edge devices. These results provide important support for the practical deployment of the model in agricultural production scenarios and offer a reference for future integration and optimization in intelligent agricultural machinery systems.

**Table 5:** Comparison of detection performance between CED-YOLOv11n and classical YOLOv11n models on different computing devices.

Operational equipment	Model	Average inference speed (/FPS)
PC computer terminal	YOLOv11n	56.3
	CED-YOLOv11n	53.2
NVIDIA Jetson AGX Xavier	YOLOv11n	25.68
	CED-YOLOv11n	23.74

## 4 Discussion

To address the challenges of small fruit size, dense clustering, severe leaf occlusion, and highly variable illumination conditions in complex natural environments, this study proposes an improved model, CED-YOLOv11n, which achieves a balance between detection accuracy and real-time performance. Experimental results across multiple scenarios demonstrate that the model maintains stable detection performance under complex lighting conditions, dense fruit distributions, and varying degrees of occlusion, with a Precision of 94.6% and an mAP of 93.8%. Meanwhile, it achieves a real-time inference speed of 53.2 FPS while keeping the model size constrained to 4.96 MB. Compared with the original YOLOv11n, the proposed method achieves an improvement of approximately 3.2 percentage points in accuracy with only a marginal change in model scale, indicating that it effectively alleviates the difficulties caused by weak saliency of Ginkgo fruits and complex background interference without significantly increasing computational burden. To further validate its edge deployment performance, the improved model was deployed on the NVIDIA Jetson AGX Xavier platform for real-world testing, where it achieved an average inference speed of 23.74 FPS, fully demonstrating its lightweight advantage and feasibility for practical engineering applications.

Analysis of the performance improvements indicates that the advantages of CED-YOLOv11n stem from the synergistic optimization of CFNet, DynamicHead, and the EIoU loss function at the levels of feature extraction, feature utilization, and bounding-box regression, rather than a simple stacking of individual modules. The ablation results show that introducing CFNet alone (Model 1) increases mAP to 93.5% while reducing the number of parameters from 2.59 × 10^6^ to 2.45 × 10^6^, indicating that CFNet enhances small-object semantic representation through cascaded multi-scale feature fusion and achieves a certain degree of model lightweighting, effectively alleviating semantic loss in traditional feature pyramid networks during small-object feature propagation. After further incorporating the EIoU loss function, Model 2 achieves an mAP of 94.0% and a Precision of 92.4%, with its main advantage lying in more accurate boundary representation of adjacent fruits in clustered scenarios, thereby reducing the localization bias commonly observed in dense small-object regression with the traditional CIoU loss. The introduction of DynamicHead further improves the model’s adaptability to scale variations and appearance instability, achieving the highest Recall of 87.2% among the single-module variants, which indicates that the dynamic detection head enhances target response stability under complex occlusion conditions through joint modulation of scale-, spatial-, and task-level attention. Overall, the integration of the three modules leads to superior performance in detection accuracy, convergence stability, and generalization ability compared with any single-module configuration.

Compared with existing fruit detection studies, CED-YOLOv11n demonstrates more targeted advantages in small-object adaptability under complex natural environments. In previous work addressing greenhouse interference in clustered tomato harvesting point detection, Deng et al. (2025) developed the YOLOv8-TP model based on YOLOv8n-pose by replacing the backbone C2f module with C2f-OREPA, introducing a PSA mechanism, and adding a CGA Fusion module in the neck, enabling simultaneous detection of tomatoes and harvesting points, this model achieved an identification accuracy of 89.8% in greenhouse scenarios. For the problem of low efficiency and limited accuracy in manual identification of seedling citrus, Gao et al. (2024) constructed the YCCB-YOLO detection model, achieving an identification accuracy of 91.79% on a seedling citrus dataset, thereby providing an efficient technical solution for seedling fruit detection in complex orchard environments. To address occlusion, small objects, and efficiency bottlenecks in citrus detection under complex orchard conditions, Wang et al. (2025b) proposed a lightweight detection framework, ELD-YOLO, which enhances feature representation through edge-aware processing, balances accuracy and computational cost via a streamlined detection head, and reduces feature loss using an adaptive upsampling strategy, achieving an accuracy of 89.7% on citrus datasets. In comparison, the proposed CED-YOLOv11n achieves an accuracy of 94.6% for real-time Ginkgo fruit detection, improving by 4.8%, 2.81%, and 4.9%, respectively, over the above methods. Moreover, the proposed model targets small-scale, densely clustered, and heavily occluded Ginkgo fruits, and while maintaining model lightweightness and high inference speed, it achieves superior detection accuracy, indicating that structurally tailored improvements based on object growth characteristics are an effective approach for enhancing fruit detection performance in complex field environments.

The proposed CED-YOLOv11n model demonstrates strong detection performance and robustness; however, due to the limitations of data collection under a single region and a single fruit maturity stage, the dataset lacks sufficient scene diversity, which may introduce a certain risk of overfitting, and its generalization capability across different crops and environments still requires further validation. The proposed collaborative improvement strategy involving CFNet, DynamicHead, and the EIoU loss function shows general optimization potential for detecting small-scale, clustered, heavily occluded, and illumination-variant fruit targets. In future work, the model can be transferred to similar crops such as wild jujube, cherry, young citrus, and clustered tomato, and its generalization ability can be further evaluated on public datasets of small-object targets such as citrus and small apples. In addition, constructing large-scale multi-region and full-growth-stage datasets, combined with multimodal perception and cross-domain fine-tuning strategies, may further enhance the model’s stability, generalization ability, and cross-crop adaptability in complex orchard environments, thereby providing stronger support for precise fruit detection in complex horticultural scenarios.

## 5 Conclusion

To address the challenges of weak feature saliency, large scale variations, and severe occlusion interference of Ginkgo fruit targets in complex natural environments, and to support the development and application of automated and intelligent Ginkgo harvesting equipment. This study proposes an innovative real-time Ginkgo fruit detection model, CED-YOLOv11n, suitable for multi-scenario conditions, based on machine vision technology and the strong performance of convolutional neural networks in feature extraction and object detection tasks. Based on this study, the following conclusions can be drawn:

1. Based on YOLOv11n, the CFNet, DynamicHead, and EIoU loss function were introduced to construct the CED-YOLOv11n Ginkgo fruit detection model. Experimental results show that, compared with YOLOv11n, the proposed model improves Precision, Recall, and mAP by 3.2%, 2.8%, and 5.5%, respectively, demonstrating a significant enhancement in detection performance.
2. The Grad-CAM visualization results and recognition performance analysis indicate that the three proposed improvement strategies can effectively enhance the model’s feature extraction capability and contextual information utilization ability, significantly reduce the interference of complex backgrounds on prediction results, and thereby further improve detection accuracy while maintaining detection efficiency.
3. Comparative results with Faster R-CNN, EfficientDet, RetinaNet, DETR, and various YOLO-based models demonstrate that CED-YOLOv11n achieves the best overall performance, with a Precision of 94.6%, a Recall of 85.4%, and an mAP of 93.8%. Meanwhile, it maintains the smallest model size while achieving a significantly higher FPS, thereby meeting the requirements of edge-computing applications in complex field environments.

The proposed CED-YOLOv11n model achieves a good balance among accuracy, stability, and real-time performance in Ginkgo fruit detection, verifying the effectiveness of the improved strategies. It can meet the requirements of automatic recognition in complex natural scenarios, provide a reliable visual perception foundation for orchard operations, and offer valuable reference for fruit object detection in smart agriculture.

## Data Availability

The original contributions presented in the study are included in the article/**Supplementary Material**. Further inquiries can be directed to the corresponding author/s.
