# Supplementary material for: An improved YOLOv11n-based method for high-precision detection of ginkgo fruits in complex natural environments
Source: Front Plant Sci. 2026 Jul 6;17:1836867. doi: 10.3389/fpls.2026.1836867 (PMC13381795; doi:10.3389/fpls.2026.1836867)
Supplement: Supplementary file 1 [file Supplementaryfile1.pdf]

Figure Captions:

Fig. S1 Label distribution analysis diagram of the ginkgo fruit dataset. (a) Dataset annotation box space and scale distribution, (b) Label box parameters pairwise correlation distribution matrix.

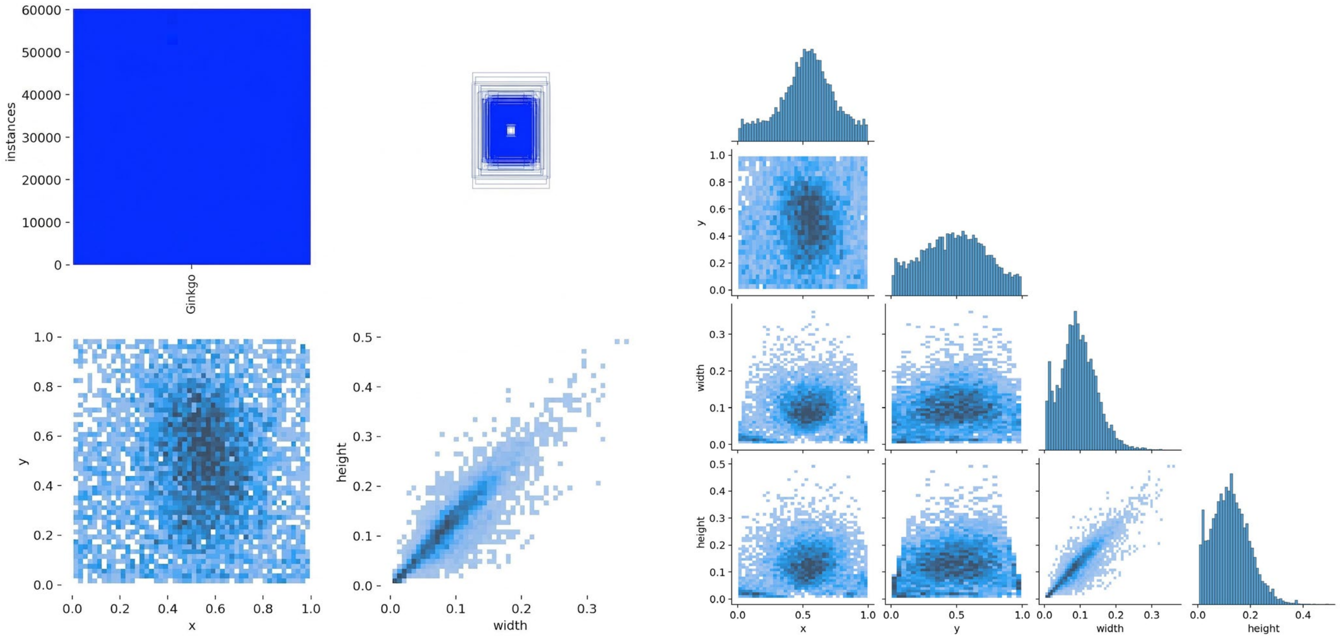

(a) (b)

Fig. S1 Label distribution analysis diagram of the ginkgo fruit dataset. (a) Dataset annotation box space and scale distribution, (b) Label box parameters pairwise correlation distribution matrix.
