# Supplementary material for: An improved YOLOv11n-based method for high-precision detection of ginkgo fruits in complex natural environments
Source: Front Plant Sci. 2026 Jul 6;17:1836867. doi: 10.3389/fpls.2026.1836867 (PMC13381795; doi:10.3389/fpls.2026.1836867)
Supplement: Supplementary file 2 [file Table1.docx]

TABLE S1 Performance evaluation results of the models on the NVIDIA Jetson AGX Xavier platform.

| **Model** | **Inference Speed (/FPS)** | | | | | | | | | | **Average** |
| --- | --- | --- | --- | --- | --- | --- | --- | --- | --- | --- | --- |
| YOLOv11n | 27.11 | 23.12 | 25.78 | 24.89 | 26.34 | 29.57 | 28.22 | 22.35 | 24.56 | 24.86 | 25.68 |
| CED-YOLOv11n | 25.34 | 27.64 | 24.11 | 22.45 | 24.22 | 20.15 | 23.11 | 25.50 | 23.56 | 21.32 | 23.74 |
